# SPINK1 promotes colorectal cancer progression by downregulating Metallothioneins expression

**DOI:** 10.1038/oncsis.2015.23

**Published:** 2015-08-10

**Authors:** R Tiwari, S K Pandey, S Goel, V Bhatia, S Shukla, X Jing, S M Dhanasekaran, B Ateeq

**Affiliations:** 1 Department of Biological Sciences and Bioengineering, Indian Institute of Technology, Kanpur, India; 2 Michigan Center for Translational Pathology, University of Michigan, Ann Arbor, MI, USA; 3 Department of Pathology, University of Michigan, Ann Arbor, MI, USA

## Abstract

Colorectal cancer (CRC) is the third most common cancer in the world, and second leading cause of cancer deaths in the US. Although, anti-EGFR therapy is commonly prescribed for CRC, patients harboring mutations in *KRAS* or *BRAF* show poor treatment response, indicating an ardent demand for new therapeutic targets discovery. SPINK1 (serine peptidase inhibitor, Kazal type 1) overexpression has been identified in many cancers including the colon, lung, breast and prostate. Our study demonstrates the functional significance of SPINK1 in CRC progression and metastases. Stable knockdown of *SPINK1* significantly decreases cell proliferation, invasion and soft agar colony formation in the colon adenocarcinoma WiDr cells. Conversely, an increase in these oncogenic phenotypes was observed on stimulation with SPINK1-enriched conditioned media (CM) in multiple benign models such as murine colonic epithelial cell lines, MSIE and YAMC (SPINK3-negative). Mechanistically, *SPINK1* promotes tumorigenic phenotype by activating phosphatidylinositol 3-kinase (PI3K/AKT) and mitogen-activated protein kinase/extracellular signal-regulated kinase (MAPK/ERK) signaling pathways, and the SPINK1-positive WiDr cells are sensitive to AKT and MEK inhibitors. Importantly, *SPINK1* silencing mediated upregulation of various Metallothionein isoforms, considered as tumor suppressors in CRC, confer sensitivity to doxorubicin, which strengthens the rationale for using the combinatorial treatment approach for the SPINK1-positive CRC patients. Furthermore, *in vivo* studies using chicken chorioallantoic membrane assay, murine xenograft studies and metastasis models further suggest a pivotal role of SPINK1 in CRC progression and metastasis. Taken together, our study demonstrates an important role for the overexpressed SPINK1 in CRC disease progression, a phenomenon that needs careful evaluation towards effective therapeutic target development.

## Introduction

Colorectal cancer (CRC) is the most frequently diagnosed cancer worldwide, nevertheless poor diagnosis of this disease still accounts for the highest number of cancer deaths globally. According to the National Institute of Health (NIH) about 132 700 new CRC cases are likely to be diagnosed this year, and 49 700 patients are estimated to succumb in the US alone. Many approved targeted therapies including monoclonal antibody (cetuximab) against epidermal growth factor receptor (EGFR) are currently utilized in treatment of metastatic CRC. Typically anti-EGFR therapy is either administered as part of first-line treatment or as a final resort when other treatments have failed. However, half of the CRC patients harboring *KRAS*, *NRAS* and *BRAF* mutations, acquire resistance to anti-EGFR drugs,^1^ highlighting the necessity for additional targeted therapies.

Previously, Cancer Outlier Profile Analysis (COPA) approach identified SPINK1 (serine peptidase inhibitor, Kazal type 1) as a high-ranking meta-outlier in a subset of prostate cancer (PCa), which demonstrates mutual exclusivity with ETS family genes expression.^2^ SPINK1, also known as pancreatic secretory trypsin inhibitor (PSTI) or tumor-associated trypsin inhibitor (TATI), encodes a 56 amino acid long peptide, is known to protect the pancreas from autodigestion by preventing premature activation of pancreatic proteases.^3^ Apart from its normal expression in pancreatic acinar cells, SPINK1 overexpression has been reported in multiple human cancers^4, 5, 6, 7, 8, 9, 10^ and increased serum SPINK1 level has been correlated with aggressive disease and poor prognosis.^4, 5, 9^ We previously demonstrated in a PCa model, the interaction between SPINK1 and EGFR, leading to receptor dimerization and phosphorylation.^11^ Furthermore, exogenous SPINK1 significantly increases cell proliferation and invasion in multiple cancers, suggesting SPINK1 as an autocrine or paracrine growth factor.^11, 12, 13, 14^ SPINK1 is also known to suppress Granzyme A-induced and serine protease-dependent cell apoptosis and confers chemoresistance to multiple drugs.^14, 15, 16^

The role of SPINK1 in stimulating mucosal repair at the site of injury and protection of the mucus layer from excessive digestion in the gastrointestinal tract has been well-established.^17^ However, elevated serum levels and tumor-specific overexpression of SPINK1 in gastric cancer and CRC respectively, are associated with advanced stage of the disease, poor prognosis and liver metastasis.^18, 19^ Many mutations in *SPINK1* have been discovered in familial pancreatitis, including the high-risk N34S haplotype, which is associated with chronic pancreatitis.^20, 21^ Interestingly, while people harboring N34S SPINK1 variant are not highly susceptible to pancreatitis than the general population, the presence of this variant significantly increases the risk of recurrent episodes.^22^ *SPINK3* (murine homolog of *SPINK1*) knockout mice died after birth due to excessive autophagy and impaired regeneration in the pancreatic acinar cells, suggesting the critical role of SPINK3 in autophagy regulation and balancing the exocrine integrity as a trypsin inhibitor.^23, 24^

Ectopic expression of SPINK1 mutant K18Y-TATI in HT-29 colon cancer cells reduces cell proliferation *in vitro* and slows down tumor growth and distant metastases to the lungs.^12^ Conversely, SPINK1 stimulates migration of the HT-29 cells in an *in vitro* wounding model of epithelial restitution assay, which was abrogated by adding neutralizing antibody against EGFR, suggesting its role in mucosal repair and intestinal injury.^25^ Currently no information is available on how SPINK1 elicits pro-invasive and pro-proliferative phenotypes in CRC, despite its critical role in stimulating mucosal repair at the site of intestinal injury. Hence, the current study aims to achieve a comprehensive understanding of the role of SPINK1 in colorectal carcinogenesis.

Our results reveal that silencing *SPINK1* in the SPINK1+ colorectal cancer line (WiDr), which also harbors *BRAF* mutation, attenuates cell invasion, proliferation, foci formation and anchorage-independent growth in soft agar assay. Conversely, exogenous addition of SPINK1-enriched media to murine colon cells increases both cell proliferation and invasion. Mechanistically, we demonstrate downregulation of AKT phosphorylation and upregulation of various isoforms of Metallothioneins (MTs), on *SPINK1* silencing. Furthermore, silencing *SPINK1* in WiDr cells confer sensitivity towards chemotherapeutic drugs by upregulating MTs. Lastly, using *in vivo* models such as chicken embryo chorioallantoic membrane assay (CAM) and murine xenograft models, we show that silencing *SPINK1* expression affected intravasation of cancer cells, tumor growth and distant metastases. Together our findings illustrate the functional significance of SPINK1 in CRC and warrants further investigations to evaluate its effectiveness as a therapeutic target in a SPINK1+ subset of CRC patients. Specifically, SPINK1 blockade may be a viable option in CRC patients, who harbor *KRAS*, *NRAS* and *BRAF* mutations, and are resistant to conventional anti-EGFR therapies.

## Results

### SPINK1 is overexpressed in colon adenocarcinoma

**Figure 1:**
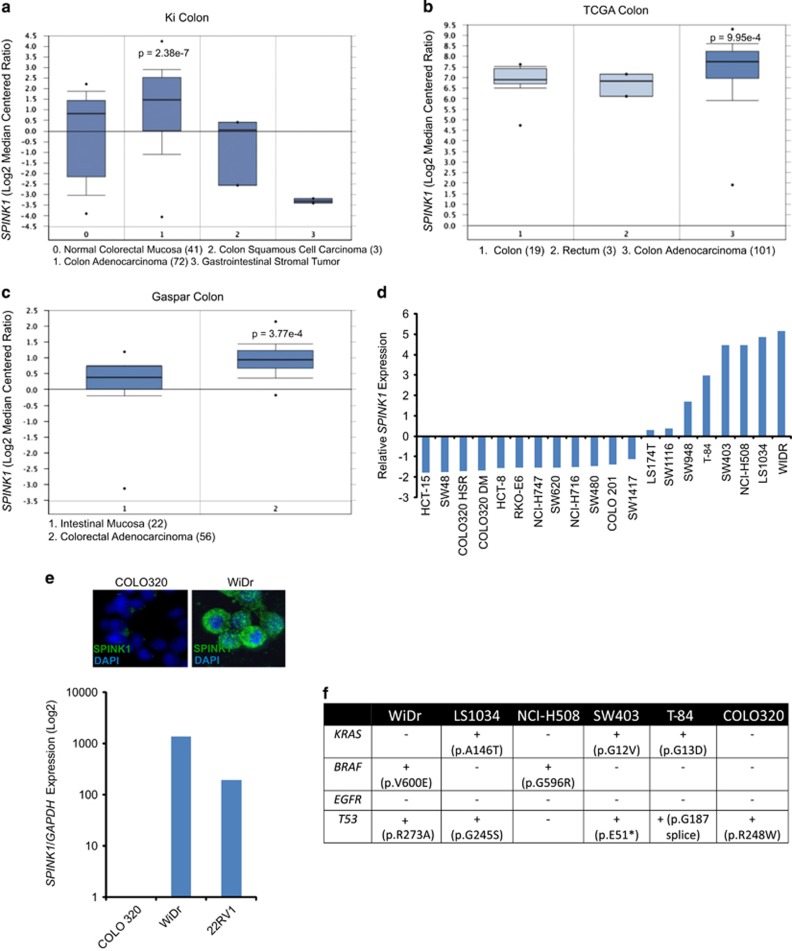
*SPINK1* is highly expressed in colon adenocarcinoma. (**a**–**c**) *SPINK1* expression in colon adenocarcinoma in three independent cohorts (Ki colon, TCGA study and Gasper colon) from Oncomine database. (**d**) Relative expression of *SPINK1* in a panel of colorectal cell lines (*n*=20). (**e**) Expression of *SPINK1* in WiDr cells and 22RV1 prostate cancer cells (SPINK1 positive) as compared with another colorectal cell line COLO320 (SPINK1 negative) measured by immunofluorescence (upper panel) and quantitative PCR (lower panel). (**f**) Mutation status of *KRAS*, *BRAF*, *EGFR* and *TP53* in SPINK1-positive colon cancer cell lines.

We queried the Oncomine database^26^ (http://oncomine.com) for publically available CRC microarray data and identified overexpression of *SPINK1* in three independent CRC data sets, namely, Ki *et al.*,^27^ Gaspar *et al.*^28^ and the TCGA study.^29^ In Ki *et al.* data set while we observed significant *SPINK1* upregulation (*P*=2.38e−7) in colon adenocarcinoma, interestingly the squamous-cell carcinomas showed downregulation (Figure 1a). Analysis of other independent data sets generated using TCGA and Gaspar *et al.*^28^ studies further supported *SPINK1* overexpression in colorectal adenocarcinoma relative to intestinal mucosa and normal colon (*P*=9.95e−4 and *P*=3.7e−4, respectively) (Figures 1b and c). We next assessed *SPINK1* expression across various colon adenocarcinoma cell line models (*n*=20) available at Oncomine. This identified WiDr cell line has the highest *SPINK1* expression followed by LS1034, NCI-H508, SW403 and T-84 (Figure 1d). The expression of SPINK1 protein in WiDr cells was confirmed by immunofluorescence, and mRNA transcript expression was comparable to the well-studied *SPINK1* outlier PCa cell line namely 22RV1 (Figure 1e). We utilized CRC cell line COLO320 as our negative control, which showed lack of both SPINK1 protein and mRNA transcript expression (Figure 1e). We next investigated the mutation status of *KRAS*, *BRAF*, *EGFR* and *TP53* in SPINK1-positive cell lines (WiDr, LS1034, NCI-H508, SW403 and T-84) and SPINK1-negative COLO320 cells using information available at the Broad-Novartis Cancer Cell Line Encyclopedia (http://www.broadinstitute.org/ccle/home). Interestingly, all SPINK1-positive CRC cell lines harbor mutations in either *KRAS* or *BRAF*, whereas mutations in *TP53* gene were present in all the cell lines except NCI-H508 irrespective of the *SPINK1* status (Figure 1f).

### SPINK1 is involved in cell proliferation and invasion in colorectal cancer

**Figure 2:**
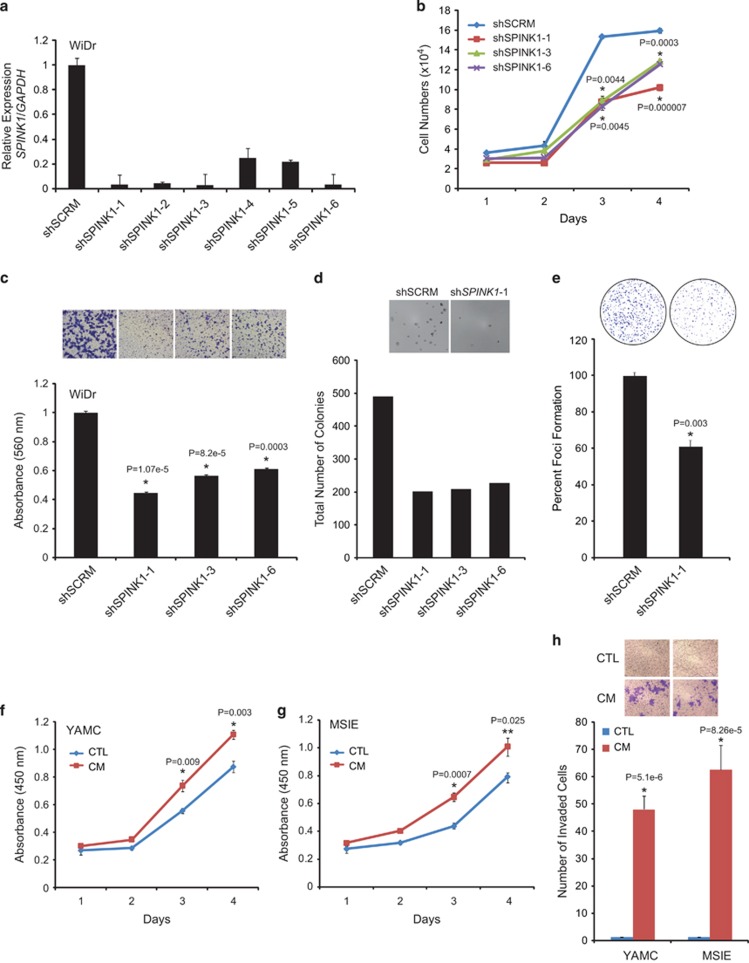
Knockdown of *SPINK1* leads to decrease in cell proliferation, invasion and anchorage-independent growth. (**a**) *SPINK1* expression in *SPINK1* knockdown WiDr cells (shSPINK1-1, shSPINK1-2, shSPINK1-3, shSPINK1-4, shSPINK1-5 and shSPINK1-6) as compared with shSCRM (Scrambled) WiDr cells. (**b**) Cell proliferation assay using sh*SPINK1*-1, shSPINK1-3, shSPINK1-6 and shSCRM WiDr cells at the indicated time points. (**c**) Boyden chamber matrigel invasion assay using shSPINK1-1, shSPINK1-3, shSPINK1-6 and shSCRM WiDr cells. (**d**) Same experimental cell lines as **c**, except soft agar assay for anchorage-independent growth. (**e**) Foci formation assay using shSPINK1-1 and shSCRM WiDr cells. (**f**) SPINK1-enriched conditioned media (CM) stimulated cell proliferation in YAMC cells measured by colorimetric water-soluble tetrazolium (WST) assay at the indicated time points. (**g**) Same as **f**, except MSIE cells were used. (**h**) Cell invasion measured by Boyden chamber matrigel invasion assay using YAMC and MSIE cells. Error bars represent mean±s.e.m. *P*-values derived from two-sided Student's *t*-test, *P<0.005, **P<0.05.

To investigate the role of SPINK1 in colon adenocarcinoma progression, stable *SPINK1*-silenced WiDr cells (shSPINK1) were generated using lentivirus-based *SPINK1* short-hairpin RNA. Knockdown efficiency of *SPINK1* in six different batches of pooled shSPINK1 cells was compared with non-targeting shSCRAMBLE (shSCRM) control cells. Pooled shSPINK1-1, shSPINK1-2, shSPINK1-3 and shSPINK1-6 cells demonstrated >80% knockdown of *SPINK1* mRNA by quantitative PCR (qPCR; Figure 2a). Next, to investigate the role of SPINK1 in cell proliferation, we performed assays using pooled shSPINK1-1, shSPINK1-3, shSPINK1-6 (showing maximum *SPINK1* knockdown) and control shSCRM cells. All three stable *SPINK1* knockdown cell lines showed significantly decreased proliferation compared with shSCRM cells (Figures 2b; *P*=0.0003; *P*=7e−6).

To confirm the role of *SPINK1* in cell invasion, Boyden chamber matrigel assay (Corning, NY, USA) was performed using shSPINK1-1, shSPINK1-3, shSPINK1-6 and control shSCRM cells. As anticipated, *SPINK1*-silenced cells (shSPINK1-1) showed decrease in cell invasion by >50% as compared with shSCRM cells (Figures 2c; *P*=1.07e-5). Furthermore, shSPINK1 cells showed decreased colonies in soft agar (Figure 2d) and foci formation (Figure 2e; *P*=0.003) when compared with shSCRM cells. To further explore the effect of exogenous SPINK1 in normal colon cells, we treated immortalized murine colon epithelial cells, MSIE and YAMC (SPINK1-negative) with SPINK1-enriched CM collected from SPINK1-positive WiDr cells and performed cell proliferation and invasion assay. CM treatment of both YAMC and MSIE cell lines resulted in significant increase in cell proliferation (Figures 2f and g; *P*=0.003 and *P*=0.025 at day 4, respectively) and invasion (Figures 2h; *P*=5.1e−6 and *P*=8.2e−5 respectively). Taken together, these findings demonstrate that endogenous or exogenous presence of SPINK1 has an important role in cell proliferation and invasion during colon adenocarcinoma progression.

### SPINK1 promotes cell proliferation by activating PI3K/AKT and MEK/ERK signaling

**Figure 3:**
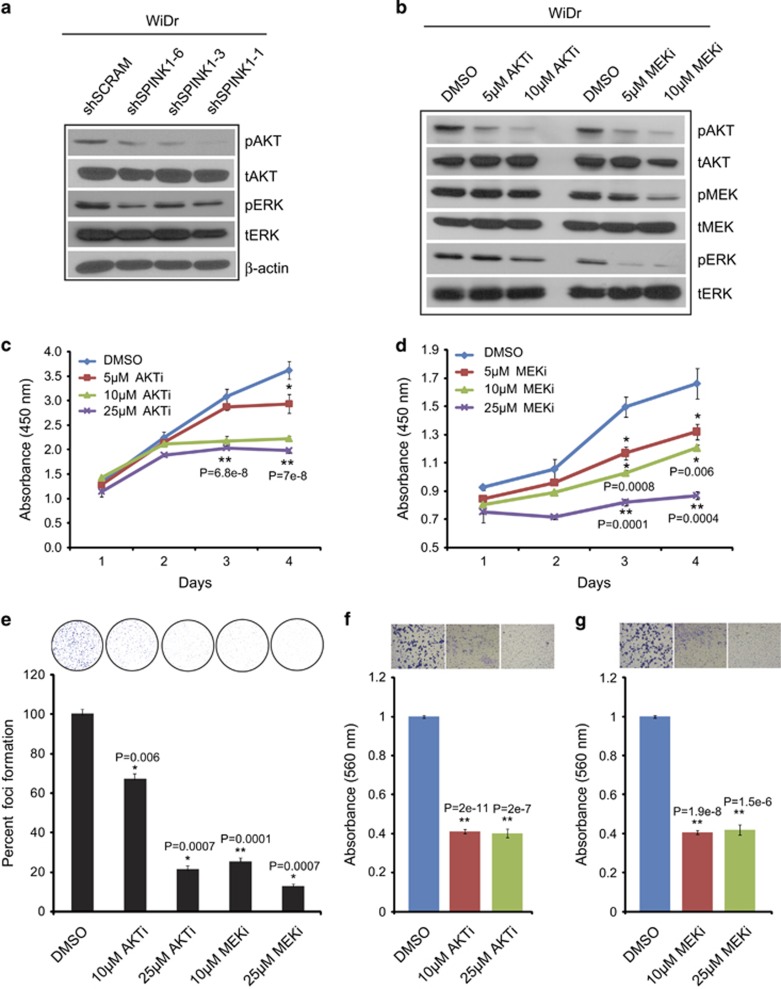
Knockdown of *SPINK1* reduces PI3K/AKT and MEK/ERK signaling. (**a**) AKT and ERK phosphorylation was determined by western blotting using shSPINK1-1, shSPINK1-2, shSPINK1-3 and shSCRM WiDr cells. (**b**) Western blotting for AKT, MEK and ERK phosphorylation for WiDr cells treated with 5 and 10 μM of AKT inhibitor (LY294002) and MEK inhibitor (PD98059) and DMSO control. (**c**) Cell proliferation assay using WiDr cells treated with 5 , 10 and 25 μM of AKT inhibitor (LY294002) with DMSO as control. (**d**) Same as **c**, except MEK inhibitor (PD98059) was used. (**e**) Foci formation assay using WiDr cells treated with AKT and MEK inhibitor. (**f**) Boyden chamber matrigel invasion assay using WiDr cells treated with 10 and 25 μM of AKT inhibitor (LY294002) and DMSO. (**g**) Same as **f**, except cells were treated with MEK inhibitor (PD98059). Error bars represent mean±s.e.m. *P*-values derived from two-sided Student's *t*-test, *P<0.05, **P<0.0005.

Phosphatidylinositol 3-kinase (PI3K/AKT) and mitogen-activated protein kinase/extracellular signal-regulated kinase (MAPK/ERK) signaling pathways are known to drive cell proliferation, survival and invasion.^30, 31^ Hence, to further elucidate the role of SPINK1 in colon cancer cell proliferation and invasion, we performed immunoblotting for pMEK (phosphorylated MAPKs), pERK (phosphorylated ERK) and pAKT (phosphorylated AKT) in stable *SPINK1* knockdown WiDr cells. We observed significant decrease in pERK and pAKT in shSPINK1-1, shSPINK1-3 and shSPINK1-6 cells as compared with shSCRM cells (Figure 3a). Next, we tested the effect of AKT inhibitor (AKTi) LY294002 and MEK inhibitor (MEKi) PD98059 (5 and 10 μM) on PI3K/AKT and MAPK/ERK pathways, respectively, using WiDr cells, as anticipated a dose-dependent decrease in pAKT, pMEK and pERK was observed (Figure 3b), suggesting that MEKi and AKTi could be used for abrogating SPINK1-mediated carcinogenesis. Further, we sought to determine whether blocking PI3K/AKT and MEK/ERK signaling in SPINK1+ colon cancer cells could hamper SPINK1-mediated oncogenic effects. AKTi and MEKi significantly inhibited WiDr cell proliferation by 44 and 48%, respectively (at higher concentration), when compared with controls (Figures 3c and d; *P*=7e−8 and *P*=0.0004, respectively). Foci assay data revealed marked decrease in the number of foci (~80% reduction at higher concentration) in the AKTi- and MEKi-treated WiDr cells as compared with controls (Figure 3e; *P*=0.0007 for both inhibitors). Likewise, significant reduction in cell invasion in Boyden chamber matrigel invasion assay was observed on AKTi and MEKi treatment (Figures 3f and g; *P*=2e−7 and *P*=1.5e−6).

### Knockdown of *SPINK1* renders MTs upregulation and sensitivity to chemotherapeutic drugs

**Figure 4:**
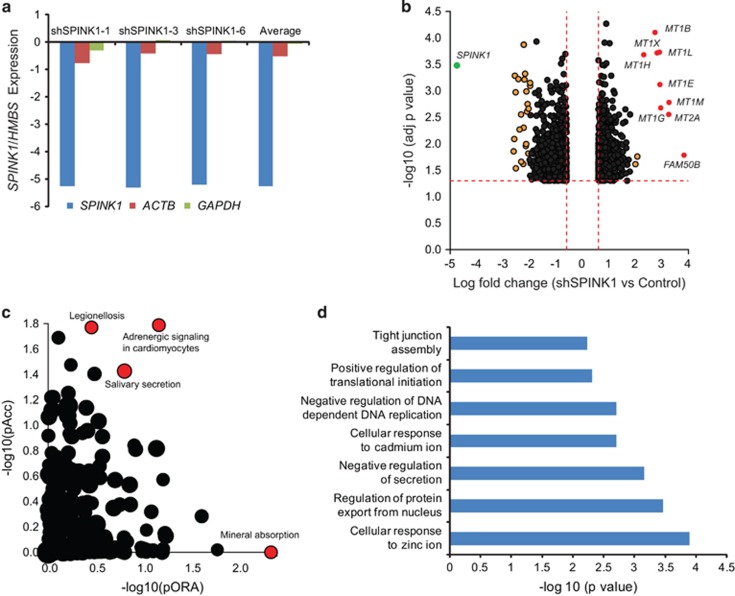
Knockdown of *SPINK1* leads to upregulation of Metallothioneins (MTs) and other important pathways. (**a**) *SPINK1* expression derived from the microarray profiling data using *SPINK1* knockdown WiDr cells, shSPINK1-1, shSPINK1-3 and shSPINK1-6 as compared with shSCRM cells. *ACTB* and *GAPDH* were used as control. (**b**) Volcano plot depicting differentially regulated genes by clustering based on probes that were enriched or depleted (*P*<0.01) in shSPINK1 cells as compared with shSCRM cells. Note: upregulation of several isoforms of Metallothioneins on *SPINK1* silencing. (**c**) Enriched cellular pathways using differentially regulated genes (*P*<0.05) uploaded onto Ingenuity pathway analysis software. (**d**) DAVID analysis showing different pathways upregulated in *SPINK1*-silenced cells.

Next, to investigate the *SPINK1*-mediated global gene-expression profiles in colon adenocarcinoma, microarray analysis was performed using shSPINK1-1, shSPINK1-3, shSPINK1-6 and shSCRM cells on Agilent Human Whole Genome Oligo Expression Arrays (Agilent Technologies, Santa Clara, CA, USA). The differentially regulated genes (*n*=1996) were visualized using volcano plot by clustering based on probes that were enriched or depleted (*P*<0.01) in shSPINK1 cells as compared with shSCRM cells. Interestingly, *SPINK1* knockdown in WiDr cells significantly upregulated *MT* family members (*MT1B*, *MT1X*, *MT1H*, *MT1L*, *MT1E*, *MT1M*, *MT2A* and *MT1G*) (Figures 4a and b). Upregulation of the *MT* family members was confirmed by qPCR using shSPINK1-1 and shSCRM cells (Supplementary Figure 1). Next, to identify cellular pathways that are enriched among the differentially regulated genes (*P*<0.05), we used multi-experiment viewer (MEV, Dana-Farber Cancer Institute, Boston, MA, USA) and uploaded the gene list into Ingenuity pathway analysis software (Qiagen, Redwood City, CA, USA). Interestingly, we found four enriched pathways, namely mineral absorption, adrenergic signaling in cardiomyocytes, salivary secretion and legionellosis to be highly significant in shSPINK1 group as compared with shSCRM cells (Figure 4c). Next, Gene Ontology term analysis using both DAVID (Database for Annotation, Visualization and Integrated Discovery, National Cancer Institute, Frederick, MD, USA) and MEV showed seven overlapping terms with high significance, which include cellular responses to zinc and cadmium ions, regulation of protein export from nucleus among others (Figure 4d). Taken together, our data suggests that SPINK1 may have a role in regulating MT expression, whose *MT* downregulation is considered as an early step in CRC progression and patients with lower *MT* expression have worse prognosis.^32, 33, 34, 35^

**Figure 5:**
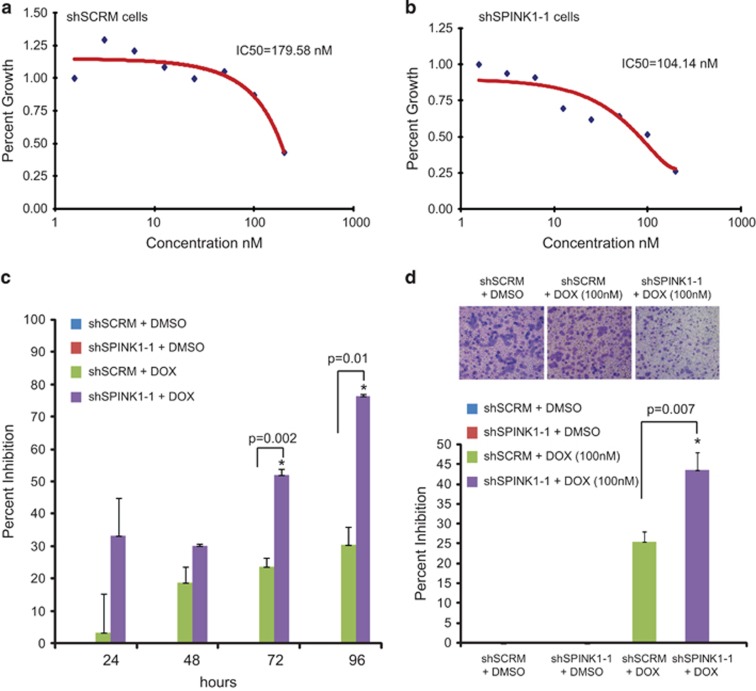
*SPINK1* knockdown in colon cancer cells confer sensitivity to chemotherapeutic drugs. (**a** and **b**) *In vitro* doxorubicin-induced cytotoxicity assay using shSPINK1-1 and shSCRM colon cancer cells. Cells were cultured in the presence of doxorubicin as shown, followed by colorimetric WST assay. IC_50_ value was calculated by automated fitting of dose–response curves using Logit regression analysis. (**c**) Percent inhibition in cell proliferation with doxorubicin treatment using shSPINK1-1 and shSCRM WiDr cells. (**d**) Boyden chamber Matrigel invasion assay using shSPINK1-1 and shSCRM WiDr cells treated with doxorubicin (100 nM). Error bars represent mean±s.e.m. *P*-values derived from two-sided Student's *t*-test, *P<0.05.

Next, we investigated whether alterations in SPINK1 expression confer sensitivity to chemotherapeutic drugs, thus we performed growth inhibition assay using shSPINK1-1 and shSCRM cells under doxorubicin treatment in dose–response curves. Interestingly, shSPINK1-1 cells were found to be more sensitive to doxorubicin (IC_50_=104 nM) than shSCRM control cells (IC_50_=179 nM) (Figures 5a and b). Next, we tested the relative effect of doxorubicin treatment on the proliferation and invasion of shSPINK1 and shSCRM cells. Treatment with doxorubicin showed 52 and 76% inhibition in shSPINK1-1 cells proliferation as compared with only 23 and 30% inhibition in shSCRM cells at 72 h and 96 h, respectively (Figure 5c; *P*=0.002 and *P*=0.01, respectively). Likewise, percent inhibition in cell invasion with doxorubicin treatment was higher (44% *P*=0.007) in shSPINK1-1 cells than inhibition (~25%) in shSCRM cells (Figure 5d). A previous study noted that *MT1G* overexpression sensitizes colorectal cells to the chemotherapeutic drugs oxaliplatin and 5-fluorouracil by activating p53 through zinc exchange and repressing NF-κB activity.^36^ Hence our data suggest a possible link between *SPINK1* knockdown, overexpression of MT1G and sensitivity to chemotherapy. Collectively these results suggest that *SPINK1* silencing (potentially involving MTs overexpression) confers sensitivity to doxorubicin in colon cancer cells, which strengthen the rationale for using the combinatorial treatment approach for the SPINK1+ CRC patients.

### Knockdown of *SPINK1* reduces tumor growth, intravasation and metastases to the lungs

**Figure 6:**
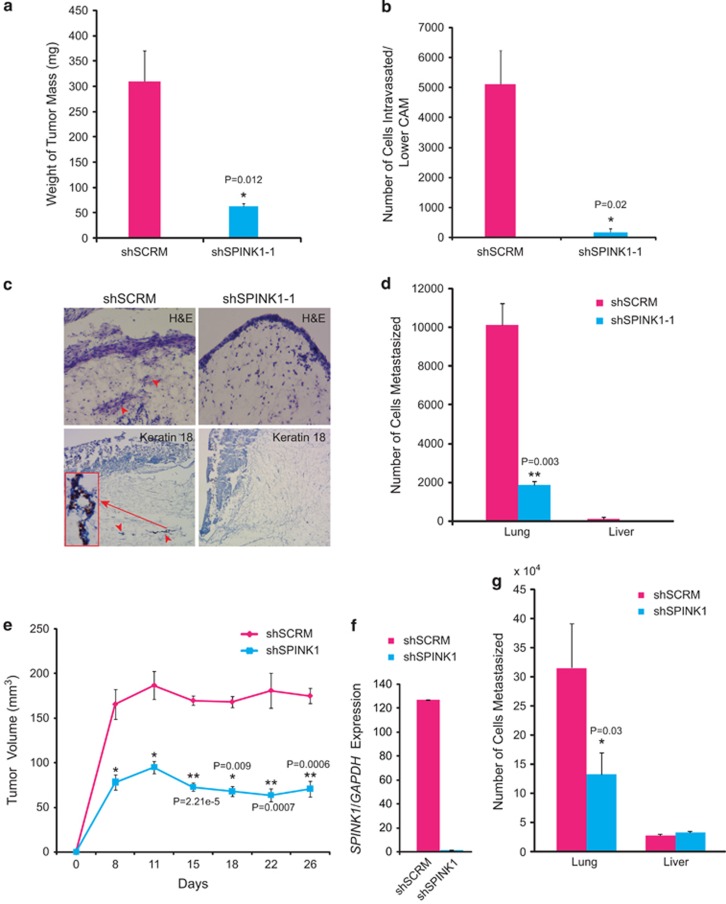
Knockdown of *SPINK1* reduces cell intravasation, tumor growth and the lung metastases. (**a**) Mean weight of the tumor mass collected from pre-fertilized eggs (*n*=8) implanted with shSCRM and shSPINK1-1 cells. (**b**) Genomic DNA extracted from the lower CAM was used to measure the intravasated human cells by qPCR using human-specific *Alu* primers. (**c**) Upper CAM harvested 3 days post engraftment of the shSPINK1-1 and shSCRM tumor cells, and representative images show the frozen sections stained for hematoxylin and eosin (top panel) and human-specific cytokeratin-18 by immunohistochemistry (bottom panel). (**d**) Genomic DNA extracted from the lungs and liver of the chick embryo was used to measure the intravasated shSCRM and shSPINK1-1 cells by qPCR using human-specific *Alu* primers. (**e**) Mean tumor growth in NOD/SCID mice subcutaneously implanted with shSPINK1-1 or shSCRM cells. Tumor growth was monitored weekly up to 4 weeks. (**f**) Relative *SPINK1* expression measured by qPCR in the xenografts excised from shSPINK1-1 and shSCRM control groups. (**g**) Genomic DNA extracted from the lung and liver of the tumor-bearing mice was used to measure the intravasated shSCRM and shSPINK1-1 cells by qPCR using human-specific *Alu* primers. Error bars represent mean±s.e.m. *P*-values derived from two-sided Student's *t*-test, *P<0.05, **P<0.005.

To investigate the tumorigenic potential of SPINK1 in CRC progression, we employed the chick embryo chorioallantoic membrane (CAM) model for tumor growth, to assess cell intravasation and metastases to distant organs. *SPINK1* knockdown in WiDr cells resulted in a significant reduction in tumor weight (Figure 6a; *P*=0.012), as well as decreased intravasation of shSPINK1 cells as compared with shSCRM cells (Figures 6b and c; *P*=0.02), suggesting that *SPINK1* knockdown in WiDr cells impaired their ability to invade the CAM basement membrane and resulted in significantly decreased number of intravasated cells in the lower CAM. Moreover, we also observed significant reduction in the ability of the WiDr cells to metastasize to the lungs in shSPINK1-1 group compared with control shSCRM (Figure 6d; *P*=0.003). Interestingly, both shSCRM and shSPINK1 cells did not show any liver metastasis in the CAM assay (Figure 6d). To further confirm these *in vivo* results, we used murine WiDr xenograft model for tumor growth using NOD/SCID mice. A significant reduction in tumor growth was observed in the shSPINK1 xenografts as compared with shSCRM control group (Figure 6e; ∼65% reduction, *P*=0.0006). Furthermore, *SPINK1* knockdown was confirmed in the excised tumors after terminating the experiment (Figure 6f). The lungs and livers were collected from both control and experimental mice and examined for spontaneous micrometastases by performing qPCR assay for the human *Alu*-sequences. A significant reduction in the lung metastatic burden in the sh*SPINK1* cells xenografted mice as compared with shSCRM group was observed (Figure 6g; *P*=0.03). Similar to CAM assay results, both control shSCRM and shSPINK1 cells failed to metastasize to the liver in the murine colon cancer xenograft model.

## Discussion

Our study shows that SPINK1 has an important role in CRC progression and metastases. We have demonstrated that *SPINK1* knockdown in the colorectal cancer cell line WiDr abrogated cell proliferation, invasion and anchorage-independent growth in soft agar assay. Moreover, murine colon epithelial cells (YAMC and MSIE) on stimulation with CM demonstrate significant increase in cell proliferation and invasion. Our findings also indicate that *SPINK1* silencing in WiDr cells significantly reduced the phosphorylation of AKT, a cytosolic signal transduction protein kinase that plays an important role in cell survival pathways.^37^ Moreover, inhibitors of the PI3K/AKT and MEK signaling pathways displayed reduction in AKT phosphorylation, cell proliferation and invasion. Previous studies showed that SPINK1 has structural similarity to EGF and binds to the EGFR in pancreatic^13^ and prostate cancer cells.^11^ SPINK1 promotes cell proliferation and invasion through autocrine and paracrine signaling, interacts with EGFR to activate downstream signaling and monoclonal antibodies against SPINK1 or EGFR could slow down the SPINK1+ PCa xenograft growth.^11^ *EGFR* copy number has been correlated with favorable survival in some CRC patients;^38^ *albeit* other independent studies demonstrated either worse disease-free survival^39^ or association with T3-stage of CRC without correlation to poor prognosis or overall shorter survival.^40^ Nevertheless, anti-EGFR therapies targeted against metastasized tumors, and correlation between EGFR-positive status in the primary and metastatic tumor remains unclear and varies between studies.^41, 42^ CRC tumors which harbor *KRAS* (~40%) and *BRAF* (~8%) mutations, correlate with poor prognosis and fail to respond to anti-EGFR treatment options.^43, 44, 45^ Therefore, the identification of other useful biomarkers in CRC that have a significant clinical impact and could guide the therapeutic treatment options are much warranted.

Physiologically SPINK1 is believed to have a protective role in gastric cancer, where it protects the mucosa from proteolytic degradation.^25, 46, 47, 48^ Nevertheless, SPINK1 overexpression is associated with poor survival in several cancer types.^9, 49, 50^ Intriguingly, most cancer types, SPINK1 and trypsin, are expressed simultaneously and show adverse associations to disease outcome.^9, 51^ Mutation at leucine-18 residue in the trypsin interaction site of SPINK1 reduced tumor growth, angiogenesis and the lung metastases in HT-29 5M21 colon cancer xenografts, suggesting that the cancer-associated SPINK1 phenotype may be related to its anti-proteinase activity.^12^ However, as reported previously we were unable to observe any effect of SPINK1 on proteases, such as trypsin or prostate-specific antigen (PSA).^11^ There have been several reports of mutations in *SPINK1* in patients with alcohol-induced chronic pancreatitis and idiopathic chronic pancreatitis, indicating *SPINK1* as a susceptible gene for chronic pancreatitis.^20, 21, 52^ Interestingly, knockout mice for *SPINK3* demonstrate lack of normal pancreas and were unable to survive beyond 15 days, whereas transgenic mice overexpressing *PSTl* (*SPINK1*) into acinar cells showed protection against caerulein-induced pancreatitis.^53^ Furthermore, high SPINK1 expression has been associated with the liver metastasis and as an independent predictor of poor prognosis and decreased overall survival in patients with CRC.^19^ Interestingly, our CAM assay and murine xenograft models for CRC spontaneous metastases experiments showed that WiDr colon cancer cells preferably metastasize to the lungs, and were undetectable in the liver tissue by human *Alu*-specific qPCR, indicating that tumor proximity to the metastatic sites may be one of the determining factors in these experiments.

MTs are a family of cysteine-rich low molecular-weight proteins, which play a critical role in zinc homeostasis, protection against heavy metals, anti-inflammatory reactions, immunomodulation and reactive oxygen species scavengers.^54, 55^ Normal-appearing mucosa from healthy individual and CRC patients showed high expression of MTs, whereas cells at the lower basal layers of the crypt exhibit negative staining for MTs,^35, 56^ suggesting that MT downregulation is an early step in CRC progression. Moreover, *MT1F*, *MT1G*, *MT1X* and *MT2A* gene expressions were significantly downregulated in CRC tissues.^57, 58^ In our current study, we found significant upregulation of the MT family members (*MT1B*, *MT1X*, *MT1H*, *MT1L*, *MT1E*, *MT1M*, *MT2A* and *MT1G*) on *SPINK1* knockdown in colon cancer WiDr cells, which corroborates with other studies that downregulation of MTs is an early indicator of CRC progression. Moreover, *MT* knockout mice are found to be more susceptible to chemically induced carcinogenesis than their wild-type counterparts^59^ and demonstrate lower disease activity index in dextran sulfate sodium-induced colitis mice model.^60^ On the other note, azoxymethane and dextran sulfate sodium-induced colon cancer mouse model showed overexpression of *SPINK3*, however, tumor burden was significantly less in *SPINK3* heterozygous mice as compared with wild-type.^61^ Many independent studies have shown that the promoter region of *MT1G,* a tumor suppressor gene, has been hypermethylated in papillary thyroid carcinoma, hepatoblastoma and esophageal squamous-cell carcinoma.^62, 63, 64^ Conversely, *MT1G* overexpression sensitizes colorectal cells to the chemotherapeutic drugs oxaliplatin and 5-fluorouracil by activating p53 through zinc exchange and repressing NF-κB activity.^36^ Our study also corroborates with this finding, wherein upregulation of MT isoforms in SPINK1-silenced cells confer sensitivity to chemotherapeutic drug doxorubicin as compared with SPINK1+ colon cancer cells. Moreover, we also found that *SPINK1*-silenced colon cancer cells perform better to doxorubicin treatment in the cell proliferation and invasion assay.

Taken together, these findings suggest protective role of the endogenous MTs against intestinal inflammation. Interestingly, our data show upregulation of *MT1B*, *MT1X*, *MT1G*, *MT1H*, *MT1E*, *MT1L*, *MT1M* and *MT2A* on *SPINK1* knockdown, which suggests dual role of SPINK1 in CRC progression. However, in CRC initiation whether upregulation of SPINK1 takes place first or downregulation of MTs, which one is the important step for triggering tumor initiation and later progression is an important question that remains to be elucidated. Nevertheless, disappointing results from the clinical trials of EGFR-targeted therapies for CRC raise uncertainties about the significance of the EGFR signaling pathway in the patients specifically harboring *KRAS*, *NRAS* and *BRAF* mutations. Therefore, our study provides strong rationale for using SPINK1 as a potential therapeutic target, whereby using SPINK1-neutralizing monoclonal antibodies or siRNA-mediated *SPINK1* silencing would abrogate SPINK1-mediated oncogenic effects in CRC, and concurrently upregulate the expression of tumor suppressor MTs in SPINK1-positive CRC patients.

## Materials and methods

### Oncomine gene-expression data sets

We used publicly available gene-expression data obtained from the TCGA study,^29^ Ki *et al.*^27^ and Gaspar *et al.*^28^ publications available on the Oncomine database.^26^ Ki *et al.*^27^ data set contained expression for 9256 transcripts across 123 specimens comprising normal colon mucosa (*n*=41), colon adenocarcinoma (*n*=72), squamous-cell carcinoma (*n*=3) and gastrointestinal-stromal tumors (*n*=2). Gaspar *et al.* data set contained 10 439 transcripts across 78 samples (intestinal mucosa, *n*=22; colorectal adenocarcinoma, *n*=56). TCGA data set contained 20 423 transcripts comprising normal colon (*n*=19), rectum (*n*=3) and colon adenocarcinoma (*n*=101) specimens.

### Cell lines and SPINK1 knockdown

CRC cell lines WiDr and COLO320-HSR were grown as per American Type Culture Collection (Manassas, VA, USA) specifications. WiDr cells were obtained from Dr Eric R Fearon's laboratory at the University of Michigan, Ann Arbor, MI, USA. Conditionally immortalized mouse colonocytes, YAMC and MSIE cells were obtained from RH Whitehead, Ludwig Cancer Institute (Melbourne, Victoria, Australia) and cultured as previously described.^65^ For stable knockdown of *SPINK1*, human lentiviral short-hairpin RNA-targeting *SPINK1* (shSPINK1) or non-silencing scrambled short-hairpin RNA (shSCRM) cloned in pGIPZ vectors were purchased from Open Biosystems (Thermo Scientific Open Biosystems, Huntsville, AL, USA) and packaged using ViraPower Lentiviral Expression System (Invitrogen, Carlsbad, CA, USA) as per manufacturer's instructions.

### Quantitative PCR

Briefly, qPCR reactions were performed with SYBR Green Master Mix (Applied Biosystems, Foster City, CA, USA) on the StepOne Plus (Applied Biosystems) using established protocol.^66^ Primer sequences are listed in the supplementary table 1.

### Cell proliferation assay

Proliferation for control and experimental cells was measured by using cell proliferation reagent WST-1 (Roche Diagnostics, Indianapolis, IN, USA) or on a Coulter counter (Beckman Coulter, Brea, CA, USA) as described previously.^11^

### Foci formation assay

Both control and experimental cells (2 × 10^3^) were plated in six-well culture dishes in prescribed medium with 5% heat-inactivated fetal bovine serum (Invitrogen). AKT (LY294002) and MEK (PD98059) inhibitors or DMSO as control was added to the culture media and replenished every third day. After 3 weeks, the assay was terminated and processed as described previously.^67^

### Basement membrane matrix invasion assay

Control and experimental cells (100 000) were seeded onto the Matrigel (BD Biosciences, San Jose, CA, USA) coated basement membrane matrix of the Boyden chamber inserts (Corning) and the assay was performed as described previously.^11^

### Soft agar colony assay

For *in vitro* anchorage-independent growth in soft agar, stable shSCRM and shSPINK1 cell suspensions (1 × 10^4^ cells) were used as previously described. Soft agar assay plates were incubated for 20 days at 37 ^o^C and colonies >40 μm were counted.

### Immunofluorescence staining

Cells were grown in a chamber slide, staining was performed using mouse anti-SPINK1 antibody (H00006690-M01; Abnova, Taipei, Taiwan) and secondary antibody (anti-mouse Alexa 555; Cell Signaling, Beverly, MA, USA) as previously described.^11^ Images were captured using a Zeiss Microscope (Carl Zeiss, Oberkochen, Germany) equipped with a high resolution CCD camera.

### Western blot analysis

Cell lysates were prepared in radioimmunoprecipitation assay (RIPA) lysis buffer, supplemented with complete proteinase (Roche, Basel, Switzerland) and phosphatase inhibitors mixture (Calbiochem, Darmstadt, Germany). Western blotting was performed using anti-phospho-MEK or -ERK or -AKT antibodies or total-MEK or -ERK or -AKT and β-actin antibodies (Cell Signaling) as previously described.^66^ The signals were visualized by enhanced chemiluminescence system as described by the manufacturer (GE Healthcare, Little Chalfont, UK).

### Gene expression profiling

Expression profiling was performed using Agilent Whole Human Genome Oligo Microarray according to the manufacturer's protocol. A total of three microarray hybridizations were performed using each stable shSPINK1 cell line sample against control shSCRM cells. Over- and underexpressed gene sets were generated by filtering to include only features with twofold average over- or underexpression (Log ratio with *P*<0.01) in all hybridization and were analyzed for enrichment of biological themes using DAVID bioinformatics platform.^68^ Gene Ontology term Bio-Process was employed to demonstrate the significantly enriched Gene Ontology terms (Biological process). Top most significant terms were plotted after converting *P*-values to −log 10 scales. Expression values of *SPINK1*, *ACTB* and *GAPDH* in shSPINK1 cells were normalized using *HMBS* expression values and plotted to show the specific downregulation of SPINK1. Significantly differentially regulated genes after employing *t*-test (*n*=1996 genes) were depicted in the volcano plot. The *P*-values were adjusted using false discovery rate correction.

### CAM assay

The CAM assay for intravasation, metastasis and tumor (xenograft) formation was performed as previously described.^66^ For xenograft growth assay with shSCRM and sh*SPINK1* WiDr cells, the embryos were killed on day 18 and the extra-embryonic xenografts were excised and weighed. The upper CAMs were processed for hematoxylin and eosin and immunostained for human cytokeratin-18 as previously described.^66^ Genomic DNA from lower CAM, the lungs and livers were prepared using Phenol/chloroform method and quantified using human *Alu*-PCR as described previously.^69^

### Mouse xenograft experiment

Five-week-old male NOD/SCID mice (Jackson Laboratory, Bar Harbor, ME, USA) anesthetized using ketamine (80 mg/kg, Intra-peritoneal), shSCRM and shSPINK1-1 cells (5 × 10^6^) were suspended in 100 μl of saline with 30% Matrigel and implanted s.c. into the dorsal flank on both sides of the mice (*n*=6 and *n*=7, respectively). Tumor growth was recorded weekly using digital calipers and tumor volumes were estimated using the formula (*π*/6) (*L* × *W*^2^), (*L*=length; *W*=width). Spontaneous metastasis in the lung and liver specimens was analyzed by performing qPCR for human *Alu*-sequences as previously described.^66^ All procedures involving mice were approved by the Committee for the Purpose of Control and Supervision of Experiments on Animals (CPCSEA) and conform to all regulatory standards.

### Statistical analysis

All values presented in the study were expressed as mean±s.e.m. The significant differences between the experimental groups were analyzed by a Student's *t*-test and a *P*-value of <0.05 or <0.001 were considered significant.
