# Correction: SPINK1 promotes colorectal cancer progression by downregulating Metallothioneins expression

**DOI:** 10.1038/s41389-021-00305-2

**Published:** 2021-02-22

**Authors:** R. Tiwari, S. K. Pandey, S. Goel, V. Bhatia, S. Shukla, X. Jing, S. M. Dhanasekaran, B. Ateeq

**Affiliations:** 1 Department of Biological Sciences and Bioengineering, Indian Institute of Technology, Kanpur, India; 2 Michigan Center for Translational Pathology, University of Michigan, Ann Arbor, MI USA; 3 Department of Pathology, University of Michigan, Ann Arbor, MI USA

Correction to: *Oncogenesis*

10.1038/oncsis.2015.23 published online 10 August 2015

The original version of this article unfortunately contained a mistake. Following the publication of this article the authors noted that one of the representative images for the Boyden chamber invasion assay in Fig. 3f was misplaced inadvertently. A corrected figure has been provided. This correction has no impact on the conclusions drawn.

The corrected figure is given below.

**Figure:**
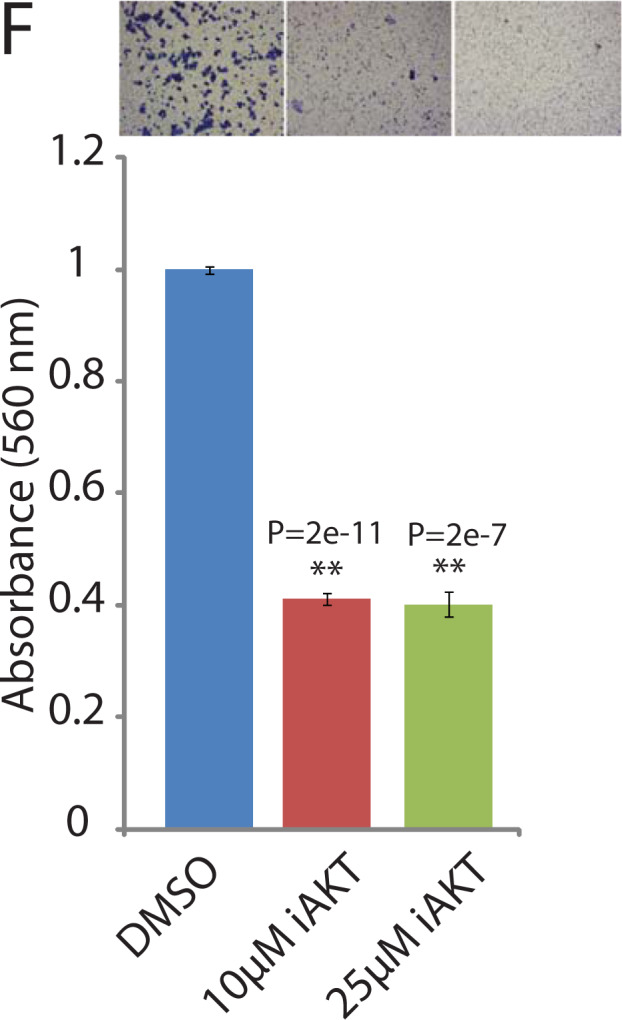
Fig. 3.

