# Supplementary material for: SPINK1 promotes colorectal cancer progression by downregulating Metallothioneins expression
Source: Oncogenesis. 2015 Aug 10;4(8):e162–. doi: 10.1038/oncsis.2015.23 (PMC4632074; doi:10.1038/oncsis.2015.23)
Supplement: Supplementary Information [file oncsis201523x1.docx]

**SUPPLEMENTARY INFORMATION**

**SPINK1 promotes Colorectal Cancer progression by down regulating Metallothioneins expression**

RitikaTiwari^1§^, Swaroop K. Pandey^1§^, Sakshi Goel^1^, Vipul Bhatia^1^, Sudhanshu Shukla^2^,

Xiaojun Jing^2^, Saravana M. Dhanasekaran^2,3^, Bushra Ateeq^1†^

^1^Department of Biological Sciences and Bioengineering, Indian Institute of Technology,

Kanpur – 208016, U.P. INDIA

^2^Michigan Center for Translational Pathology, ^3^Department of Pathology, University of Michigan, Ann Arbor, MI, 48109, USA,

^§^ These authors contributed equally to this work.

**FUNDING SUPPORT:** This work is supported by the Wellcome Trust/ DBT India Alliance grant [IA/I(S)/12/2/500635to BA] and intra-mural grant from the Indian Institute of Technology, Kanpur [IITK/BSBE/20130015 to BA].

**CONFLICT OF INTERESTS STATEMENT:** The authors declare no conflicts of interest or disclosures.

^†^**Correspondence to:**

Bushra Ateeq, Ph.D.

Department of Biological Sciences & Bioengineering

Indian Institute of Technology,

Kanpur-208016, U.P., INDIA

Phone: +91 512 2594083

Fax: +91 512 2594010

E-mail: [bushra@iitk.ac.in](mailto:bushra@iitk.ac.in)

**
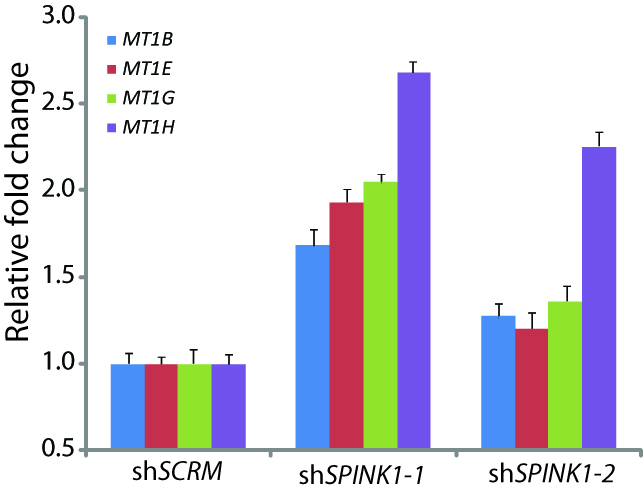
**

**Supplementary Figure 1:** Relative fold change in the expression of various metallothioneins (*MT1B*, *MT1E*, MT1G and *MT1H*) in shSPINK1-1, shSPINK1-2 as compared to shSCRM cells as determined by qRT-PCR.

| **Genes** | **Sequence (5’-3’)** |
| --- | --- |
| *SPINK1* | 5’-TGTCTGTGGGACTGATGGAA-3’  5’-AGGCCCAGATTTTTGAATGA-3’ |
| *MT1G* | 5’- GGGAACTCTAGTCTCGCCTC-3’  5’- ATTTGTACTTGGGAGCAGGG-3’ |
| *MT1B* | 5’-CTCCAGGCTTGTCTTGGCTC-3’  5’-CAGCGGCACTTCTCTGATGA-3’ |
| *MT1H* | 5’- ATCTGCAAAGGGGCGTCAGA-3’  5’- GAATGTAGCAAATGAGTCGGAGTT-3’ |
| *MT1E* | 5’- AGCATCCCCTTTGCTCGAAAT-3’  5’- CAGCTGCACTTCTCCGATG-3’ |
| *ALU* | 5’- ACGCCTGTAATCCCAGCACTT-3’  5’- TCGCCCAGGCTGGAGTGCA-3’ |
| *GAPDH* | 5’-TGCACCACCAACTGCTTAGC-3’  5’-GGCATGGACTGTGGTCATGAG-3’ |

**Supplementary Table 1:** Quantitative PCR Primers sequences used for monitoring transcript expression.
